# *HRAS* is a therapeutic target in malignant chemo-resistant adenomyoepithelioma of the breast

**DOI:** 10.1186/s13045-021-01158-3

**Published:** 2021-09-08

**Authors:** Ivan Bièche, Florence Coussy, Rania El-Botty, Sophie Vacher, Sophie Château-Joubert, Ahmed Dahmani, Elodie Montaudon, Cécile Reyes, David Gentien, Fabien Reyal, Francesco Ricci, André Nicolas, Caterina Marchio, Anne Vincent-Salomon, Marick Laé, Elisabetta Marangoni

**Affiliations:** 1 Genetics Department, Institut Curie, University of Paris, Paris, France; 2 Department of Diagnostic and Theranostic Medicine Paris, University of Paris, Paris, France; 3 INSERM U1016, Institut Cochin, Paris, France; 4 Medical Oncology Department, Institut Curie, PSL Research University, Paris, France; 5 Translational Research Department, Institut Curie, PSL Research University, 26 Rue d’Ulm, 75005 Paris, France; 6 BioPôle Alfort, Ecole Nationale Vétérinaire d’Alfort, Maisons Alfort, France; 7 Surgery Department, Institut Curie, PSL Research University, Paris, France; 8 INSERM U932, Immunity and Cancer, Institut Curie, Paris, France; 9 Pathex, Institut Curie, PSL Research University, Paris, France; 10 Institut Curie, Pathology Department, PSL Research University, Paris, France; 11 Present Address: Pathology Unit, Candiolo Cancer Institute, FPO-IRCCS, Candiolo, Italy; 12 Department of Medical Sciences, University of Turin, Turin, Italy; 13 Pathology Department, Centre Henri Becquerel, INSERM U1245, Université Rouen Normandie, Rouen, France

**Keywords:** Adenomyoepithelioma, *HRAS*, PDX, MEK inhibitor

## Abstract

Malignant adenomyoepithelioma (AME) of the breast is an exceptionally rare form of breast cancer, with a significant metastatic potential. Chemotherapy has been used in the management of advanced AME patients, however the majority of treatments are not effective. Recent studies report recurrent mutations in the *HRAS* Q61 hotspot in small series of AMEs, but there are no preclinical or clinical data showing H-Ras protein as a potential therapeutic target in malignant AMEs. We performed targeted sequencing of tumours’ samples from new series of 13 AMEs, including 9 benign and 4 malignant forms. Samples from the breast tumour and the matched axillary metastasis of one malignant *HRAS* mutated AME were engrafted and two patient-derived xenografts (PDX) were established that reproduced the typical AME morphology. The metastasis-derived PDX was treated in vivo by different chemotherapies and a combination of MEK and BRAF inhibitors (trametinib and dabrafenib). All malignant AMEs presented a recurrent mutation in the *HRAS* G13R or G12S hotspot. Mutation of *PIK3CA* were found in both benign and malignant AMEs, while *AKT1* mutations were restricted to benign AMEs. Treatment of the PDX by the MEK inhibitor trametinib, resulted in a marked anti-tumor activity, in contrast to the BRAF inhibitor and the different chemotherapies that were ineffective. Overall, these findings further expand on the genetic features of AMEs and suggest that patients carrying advanced HRAS-mutated AMEs could potentially be treated with MEK inhibitors.

**Supplementary Information:**

The online version contains supplementary material available at 10.1186/s13045-021-01158-3.

**To the Editor**,

Adenomyoepithelioma (AME) of the breast is a rare biphasic tumour of breast composed of epithelial and myoepithelial cells. It is generally a benign disease and cases of malignant AME are rare [1]. Importantly, however, metastases have been documented even in cases lacking a histologically overt malignant component [2]. The epithelial component may express estrogen receptor (ER) and progesterone receptor (PR) [1]. Given the rarity of the disease, most of the literature consists of individual case reports or studies with a few patients. A specific treatment for metastatic AME has not been determined, and the prognosis of malignant AME with distant metastases is very poor [3, 4].

In the present study we analyzed the mutational profile of 13 AMEs (9 benign and 4 malignant forms), whose histo-pathological characteristics are summarized in Table 1. These cases were diagnosed as AMEs based on the criteria defined by 2019 World Health Organization Classification of the Breast Tumours [5]. Nine AMEs (69%) expressed estrogen receptor (ER). The mutational analysis revealed recurrently mutated genes, including *HRAS* (5/13, 38%), *PIK3CA* (4/13, 31%), and *AKT1* (4/13, 31%) (Table 1). The *HRAS* mutations affected the following mutation hotspots: three p.G13R, one p.G12S and one p.Q61R hotspot mutations. Mutations in the *AKT1* gene (E17K) were exclusively found in benign ER + AMEs, while three out of four *PIK3CA* mutations (H1047R) were detected in ER-negative AMEs. *HRAS* was mutated in the four malignant AMEs (three in the G13R and one in the G12S hotspots), suggesting that these mutation hotspots may represent important driver of malignant AMEs. To our knowledge, only one case of malignant AME mutated for the *HRAS* G12 hotspot was previously identified (G12D) [6]. The low frequency of *HRAS* Q61R/K mutation hotpsot was in agreement with two studies [6, 7], while a third study published by Geyer et al. reported recurrent mutations of the *HRAS* Q61R mutation [8].

**Table 1:** clinical and pathological characteristics of AMEs

Case	Age	Size (mm)	Category	Architecture	Myoepithelial cells	Mitosis /mm2	Cytologic atypia	Necrosis	Metaplasia/ Associated findings	HER2	ER, PR	Follow-up (mo)	Recurrence/metastasis	*HRAS*	*PIK3CA*	*AKT1*
T1	64	9	Benign	Tubular	Clear	0	Mild	Present	Squamous, sebaceous	0	ER + PR +	NA	NA	Q61R	w.t	w.t
T2	53	15	Benign	Tubular	Clear	1	Mild	Absent		0	ER + PR +	NA	NA	w.t	H1047R	w.t
T3	38	24	Benign	Tubular	Clear	1	Moderate	Absent	Squamous	0	ER + PR +	NA	NA	w.t	w.t	E17K
T4	62	15	Benign	Tubular	Clear	1	Mild	Present		0	ER + PR-	15	No	w.t	w.t	w.t
T5	63	12	Benign	Tubular, lobulated, papillary	Clear	1	Mild	Absent		0	ER + PR-	36	No	w.t	w.t	E17K
T6	37	18	Benign	Tubular, lobulated, papillary, cystic	Clear	1	Mild	Absent	Squamous, chondroid and myxoid matrix	0	ER + PR-	9	No	w.t	w.t	E17K
T7	70	9	Benign	Tubular	Clear	1	Moderate	Absent		0	ER + PR-	91	Recurrence	w.t	w.t	E17K
T8	36	20	Benign	Tubular	Clear, spindle	0	Mild	Absent		0	ER- PR-	NA	NA	w.t	H1047R	w.t
T9	66	16	Benign	Tubular, lobulated	Clear	0	Mild	Absent		0	ER- PR-	6	No		w.t	w.t
T10	84	25	Malignant	Tubular, lobulated	Clear	3	Severe	Present		0	ER + PR-	12	Recurrence	G13R	w.t	w.t
T11	76	18	Malignant	Tubular, lobulated	Clear	3	Moderate	Present		0	ER- PR-	NA	NA	G13R	H1047R	w.t
T12	60	19	Malignant	Tubular, spindle, cystic	Clear, spindle	6	Severe	Present		0	ER- PR-	75	No	G13R	H1047R	w.t
T13	55	55	Malignant	Tubular	Clear	10	Severe	Absent		0	ER + PR-	11	Metastasis	G12S	w.t	w.t

*ER* estrogen receptor, *PR* progesterone receptor, *NA* not available, *w.t.* wild-type

Mutations in the *AKT1* and *PIK3CA* genes were mutual exclusive in our series, while 2 out of four malignant AMEs harboured mutations in both *HRAS* and *PIK3CA* genes. These findings are concordant with those previously reported [7, 8] and underline the co-occurrence of two cancer driver genes in a fraction of malignant AMEs.

From one of the four malignant AMEs patients (T13), whose clinical history is summarized in Fig. 1a, we could generate two PDX, HBCx-120 and HBCx-121, established from the engraftment of the breast tumour and the axillary lymph node metastasis, respectively. The histological analysis of xenografts tumors showed that tumor morphology and immunohistochemistry profile was concordant with patient’s samples (Fig. 1b). Both patient’s nodal metastasis and HBCx-121 PDX show loss of ER expression, as compared to the matched breast tumour and HBCx-120 PDX. This phenotypic discordance between the primary tumor and the metastasis is frequent in breast cancer progression and metastases, is generally associated to a worse survival and could be a consequence of intra-tumour heterogeneity and subclonal evolution of ER negative cells in the nodal metastasis [9, 10].

**Fig. 1:**
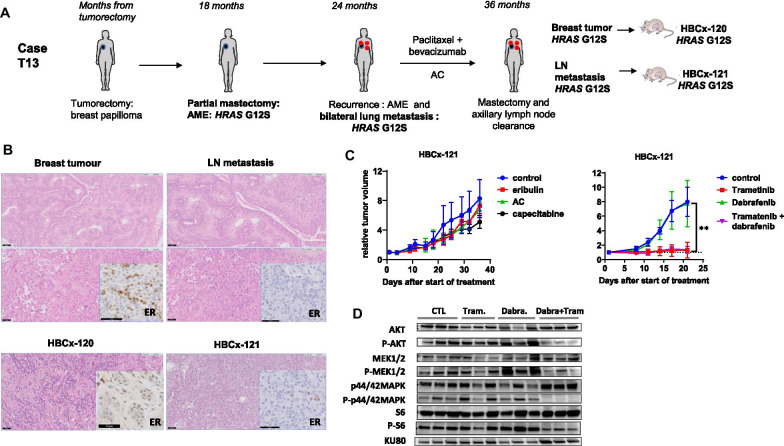
Treatment response of a PDX established from a AME patient (T13). **a** Clinical history of patient T13 and PDX establishment from the breast and the axillary lymph node tumour samples. The patient presented a mammary breast lesion initially diagnosed as atypical papilloma. The patient relapsed and underwent partial mastectomy 18 months later. The breast lesion was a benign AME, characterized by a proliferation of myoepithelial cells p63+, CD10+ around epithelium-lined spaces in a lobulated, tubular and papillary pattern. This lesion was sequenced and the *HRAS* G12S mutation was identified. Six months later, the patient presented a growing breast nodule in the same area and an axillary lymph node and bilateral lung metastases. Core needle biopsy of the breast tumour revealed a malignant AME ER positive. A biopsy of a lung metastasis was sequenced and the *HRAS* G12S mutation was identified. The patient received 6 cycles of chemotherapy with paclitaxel and bevacizumab followed by AC (Adriamycin + Cyclophosphamide). Repeat CT scans of the thorax showed progression of the lung metastases during and after chemotherapy treatment. Total mastectomy with axillary lymph node dissection after 12 months of chemotherapy was performed. The breast primary tumour was multifocal and 25% of cancer cells were ER positive. One lymph node (LN) was metastatic with capsular effraction. Samples from the breast tumor and the LN metastasis carried the *HRAS* G12S mutation and were engrafted to generate HBCx-120 and HBCx-121 PDX models, respectively. **b** histology of patients’ breast tumour and lymph node metastasis and matched PDX HBCx-120 and HBCx-121. Scale is indicated by a black bar measuring 100 µm (first row) and 50 µm (second and third rows). The breast tumour and the matched PDX HBCx-120 were ER + (25%) and PR negative. **c** Tumour growth of HBCx-121 PDX in response to different chemotherapies (eribulin, AC and capecitabine) and to the combination of trametinib with. Statistical analysis of tumour growth inhibition based on relative tumour volume was performed with the Mann–Whitney test. **d** Western Blot analysis of treated tumours showing the phosphorylation status of AKT, MEK, p44/42 MAPK (ERK) and S6. Tumours were harvested after 3 weeks of treatment and three xenografts from each treatment group were analysed

Patient’s tumour samples including the two mastectomies (partial and total), the lymph node and the lung metastasis, and PDX samples carried the *HRAS* p.Gly12Ser mutation hotspot. As *HRAS* mutations are associated to activation of RAF/MEK/ERK signaling in different cancers [11], we treated the PDX HBCx-121 by a combination of dabrafenib (a RAF inhibitor) and trametinib (a MEK1/2 inhibitor). In parallel, we determined the response to different chemotherapies: AC (Adriamycin and cyclophosphamide), capecitabine and eribulin, three standard of care currently used for breast cancer treatment. PDX HBCx-121 responded with stable disease to trametinib (tumour growth inhibition of 82%), while dabrafenib had no effect on tumor growth (Fig. 1c). The combination of trametinib with dabrafenib did not increase the anti-tumour activity, suggesting that the combination effects are mediated by the MEK inhibitor. The PDX was resistant to the three chemotherapies tested.

To our knowledge, there are no clinical nor preclinical evidence showing that patients or PDX models of *HRAS* mutated AMEs could respond to MEK inhibitors. Trametinib as a single-agent is approved for the treatment for metastatic melanoma in patients with BRAF V600E or V600K mutations [12]. Inhibition of MAPK and P-AKT signaling pathways in treated tumours was analysed by Western Blot (Fig. 1d). Phospho-p44/42 MAPK (Erk1/2) was strongly inhibited in the combination group, while in trametinib-treated tumours the inhibition was heterogeneous among the different xenografts. In tumours treated by the combination, expression of P-AKT was strongly inhibited and expression of P-S6, the downstream effector of the PI3K/AKT/mTOR pathway, was decreased. This indicates that targeting the MAPK pathway with inhibitors that act at different levels, leads to a more profound inhibition of both P-ERK and P-AKT pathways, although this was not associated to increased anti-tumour activity.

In summary, we report a new series of AMEs showing recurrent mutations in the *HRAS* G12 and G13 hotspots. The treatment of a *HRAS*-mutated AME PDX with a FDA-approved MEK inhibitor (trametinib) exhibited significant anti-tumour activity, demonstrating that *HRAS* mutation is a therapeutic target in malignant AMEs. MEK inhibitors could be an important new approach for the treatment of *HRAS* mutated AMEs patients.

## Supplementary Information

**Additional file 1**. Material and Methods and References.

## Data Availability

The datasets used and/or analyzed during the current study are available from the corresponding author on reasonable request.
