# Supplementary material for: HRAS is a therapeutic target in malignant chemo-resistant adenomyoepithelioma of the breast
Source: J Hematol Oncol. 2021 Sep 8;14:143. doi: 10.1186/s13045-021-01158-3 (PMC8424935; doi:10.1186/s13045-021-01158-3)
Supplement: Supplementary file 1 — Additional file 1. Material and Methods and References. [file 13045_2021_1158_MOESM1_ESM.docx]

**Supplementary Material for**

**HRAS is a therapeutic target in malignant chemo-resistant adenomyoepithelioma of the breast**

**This PDF file includes:**

**Material and Methods**

**References**

**Material and Methods**

**AMEs patients**

Samples from 13 AMEs excised from patients treated at Institut Curie and Centre Henri Becquerel hospitals between 1980 and 2015 were analyzed. All patients gave their consent for the use of their samples for research purposes, by signing an informed consent form.

The age at diagnosis was comprised between 36 and 84 years. The tumour size was comprised between 9 and 55 mm.

**Histological and immunohistochemistry analyses**

Formalin-fixed paraffin-embedded tissues were obtained via core needle biopsy or open biopsy/surgical excision. Representative hematoxylin and eosin–stained slides from the cases were reviewed by three breast pathologists for conﬁrmation of diagnosis based on the criteria deﬁned by 2019 World Health Organization Classiﬁcation of the Breast Tumours (1). Differential diagnosis such as intraductal papilloma, ductal adenoma, nodular adenosis, pleomorphic adenoma, adenoid cystic carcinoma, non specific carcinoma were ruled out. Assessment of growth pattern, architecture, tumour borders, mitotic activity and atypias in the two components, necrosis were evaluated. AMEs were then classiﬁed into 2 categories, benign and malignant. A tumour was referred as “malignant AME” if the tumour exhibited overgrowth of epithelial or myoepithelial component, severe cytologic atypia, inﬁltrative growth pattern, increased mitotic activity (>3/10 hpf - high power field) or necrosis.

All tumours were tested by immunohistochemistry for epithelial and myoepithelial markers (CKAE1/AE3, CD10, p63) to identify the double cell layer. Estrogen receptor (ER), progesterone receptor (PR) and HER2 expression we evaluated . The ER and PR status was defined according to the American Society of Clinical Oncology (ASCO)/College of American Pathologists (CAP) guidelines (2, 3). Cases were considered to be ER or PR positive if at least 10% of the tumour cells expressed estrogen and/or progesterone receptors (ER/PR). HER2 expression was determined by immunohistochemistry, with scoring according to the American Society of Clinical Oncology (ASCO)/College of American Pathologists (CAP) guidelines (3).

**PDX establishment and *in vivo* experiment**

Female Swiss nude mice were purchased from Charles River (Les Arbresles, France) and maintained under specific pathogen-free conditions. Their care and housing were in accordance with institutional guidelines and the rules of the French Ethics Committee (project authorization no. 02163.02). HBCx-120 and HBCx-121 PDX were established from breast cancer recurrence and synchronous lymph node metastasis, respectively, with patient’s informed consent. For PDX establishment, tumor fragments removed during patient’s surgery were grafted into the interscapular fat pad of female Swiss nude mice under anesthesia, as previously described previously described (5, 6).

HBCx-121 xenografts were treated with vehicle (0.1% Tween-20 or 0.5% hydroxypropyl methylcellulose and 0.2% Tween-80), dabrafenib (30 mg/kg per day), and trametinib (1 or 0.6 mg/kg per day) by oral gavage for 21 days. Trametinib and dabrafenib were purchased from MedchemExpress. When tumours reached a volume of 60 to 200 mm3, mice were randomly assigned to the control or treated groups. Each group of treatment consisted of eight mice.

Adriamycin (DOX, doxorubicin, Teva Pharmaceuticals, Paris, France) and cyclophosphamide (Endoxan, Baxter, Maurepas, France) were administered by the intraperitoneal (ip) route, at doses of 2 mg/kg, and 100 mg/kg, respectively, every three weeks. Capecitabine (Xeloda, Roche Laboratories, Nutley, NJ) was administered per os at a dose of 540 mg/kg/day. Eribulin was administered at 0.4 mg/kg weekly by intravenous injection.

Tumour growth was evaluated by measuring two perpendicular tumor diameters with calipers twice a week. Individual tumor volumes were calculated: V=axb2/2, where “a” is the largest diameter, “b” is the smallest diameter. For each tumour, volumes were expressed in relation to the initial volume as relative tumour volume (RTV). Tumour growth inhibition (TGI) of treated tumours versus controls was calculated as the ratio of the mean RTV (relative tumour volume) in the treated group to the mean RTV in the control group at the same time (5). Statistical significance of TGI was calculated by a paired Student’s t test by comparing tumour volumes in the treated and control groups.

**Targeted Next Generation Sequencing (NGS)**

Tumours were analyzed with two custom NGS panels: one panel that includes exons of 15 cancer associated genes showing hotspot activating mutations of interest in oncology (NGS panel used in diagnostic practice), and a second panel that includes the 91 most frequently mutated genes in breast cancer (frequencies>1%), as previously detailed (5). NGS was performed on an Illumina HiSeq2500 sequencer and the genomic variants were annotated with COSMIC and 1000 genome databases (7).

The 91 genes panel was used for the tumour that was engrafted in mice (T13) and applied to the original donor tumour and to the PDX samples.

**Western Blot analysis**

Proteins were extracted from tumours using Laemmli buffer (50 mM Tris HCL pH 8, 2 mM DTT, 2% SDS, 5% glycerol), supplemented with protease and phosphatase inhibitors. Lysates were resolved on 10% agarose gels, transferred onto nitrocellulose membranes (Bio-Rad, Hercules, CA, USA) and immunoblotted with rabbit antibodies against KU80 (Cell Signaling Technology, #2753), AKT (Cell Signaling Technology, #9272), P-AKT (Ser473) (Cell Signaling Technology, #4058), P-p44/42 MAPK (Cell Signaling Technology, #4370), p44/42 MAPK (Cell Signaling Technology, #9102), MEK1/2 (Cell Signaling Technology, #9126), P-MEK1/2 (Cell Signaling Technology, #9154), S6 (Cell Signaling Technology, #2117), P-S6 (Ser235/236) (Cell Signaling Technology, #2211). After washes, membranes were incubated with the appropriate horseradish peroxidase-conjugated affinity-purified goat anti-rabbit secondary antibodies (Jackson ImmunoResearch Laboratories, Inc., Interchim).

References

1. Tan PH, Ellis I, Allison K, Brogi E, Fox SB, Lakhani S, et al. The 2019 World Health Organization classification of tumours of the breast. Histopathology. 2020;77(2):181-5.

2. Allison KH, Hammond MEH, Dowsett M, McKernin SE, Carey LA, Fitzgibbons PL, et al. Estrogen and Progesterone Receptor Testing in Breast Cancer: ASCO/CAP Guideline Update. J Clin Oncol. 2020;38(12):1346-66.

3. Woo JW, Lee K, Chung YR, Jang MH, Ahn S, Park SY. The updated 2018 American Society of Clinical Oncology/College of American Pathologists guideline on human epidermal growth factor receptor 2 interpretation in breast cancer: comparison with previous guidelines and clinical significance of the proposed in situ hybridization groups. Hum Pathol. 2020;98:10-21.

4. Hatem R, Labiod D, Chateau-Joubert S, de Plater L, El Botty R, Vacher S, et al. Vandetanib as a potential new treatment for estrogen receptor-negative breast cancers. Int J Cancer. 2016;138(10):2510-21.

5. Coussy F, de Koning L, Lavigne M, Bernard V, Ouine B, Boulai A, et al. A large collection of integrated genomically characterized patient-derived xenografts highlighting the heterogeneity of triple-negative breast cancer. Int J Cancer. 2019.

6. Marangoni E, Vincent-Salomon A, Auger N, Degeorges A, Assayag F, de Cremoux P, et al. A new model of patient tumor-derived breast cancer xenografts for preclinical assays. Clin Cancer Res. 2007;13(13):3989-98.

7. Forbes SA, Bhamra G, Bamford S, Dawson E, Kok C, Clements J, et al. The Catalogue of Somatic Mutations in Cancer (COSMIC). Curr Protoc Hum Genet. 2008;Chapter 10:Unit 10 1.
